# Longitudinal course of eating disorders after transsexual treatment: a report of two cases

**DOI:** 10.1186/s13030-017-0118-4

**Published:** 2017-12-17

**Authors:** Maiko Hiraide, Saki Harashima, Ryo Yoneda, Makoto Otani, Mami Kayano, Kazuhiro Yoshiuchi

**Affiliations:** Department of Stress Sciences and Psychosomatic Medicine, Graduate School of Medicine, The University of Tokyo, 7-3-1 Hongo, Bunkyo-ku, Tokyo, 113-8655 Japan

**Keywords:** Gender dysphoria, Eating disorders, Gender reassignment surgery, Gender confirmation surgery

## Abstract

**Background:**

Several reports have been published on patients with gender dysphoria and eating disorders. However, there have been few reports on the longitudinal course of eating disorders after gender reassignment surgery (GRS)/gender confirmation surgery (GCS).

**Case presentation:**

We report two Japanese cases of transsexual persons with eating disorders who underwent GRS/GCS, one male-to-female (MtF) and one female-to-male (FtM). Case 1 was a 35-year MtF person who had a 14-year-course of bulimia nervosa that developed after GRS. Case 2 was a 35-year FtM person with anorexia nervosa who underwent GCS 9 years before.

**Conclusions:**

We found that the treatment of our transsexual patients influenced the course of their eating disorders for a long period, which could be attributable partly to the cultural situation in Japan, an East Asian country. It is possible that many gender identity problems and identity problems in general persist even after surgery and treatment; therefore, continual clinical support should be provided for patients with gender dysphoria and eating disorders even after hormonal therapy or GRS/GCS.

## Background

Gender dysphoria is characterized by a difference between the individual’s expressed/experienced gender and the gender that others would assign him or her [1]. Several previous case reports showed that patients with gender dysphoria had eating disorders [2–10].

One of the treatments for gender dysphoria is gender reassignment surgery (GRS)/gender confirmation surgery (GCS). Some reports have been published on the relationship between GRS/GCS and the course of eating disorders [2, 5, 6, 8, 9]. However, these previous reports followed up patients after hormonal treatment or GRS/GCS for only a few years.

Here we introduce the longitudinal course of two transsexual Japanese patients, one male-to-female (MtF) and one female-to-male (FtM), with eating disorders who underwent GRS/GCS approximately 10 years before.

Previous studies also reported that MtF people like to lose weight to get slimer, which is considered the female ideal of attractiveness, while FtM people like to lose weight to reduce their breast and hip size and to stop their menstruation [11–14]. With regard to the two patients here in presented, we identified the primary diagnosis as gender dysphoria, with a coexisting eating disorder. Our cases have body dissatisfaction or strong desire to lose weight because they desire to be closer to the “ideal gender” body as shown in previous studies. We also confirmed that they did not meet the criteria for personality disorders, anxiety disorders, or autism spectrum disorders.

In addition, there were fewer reports on FtM patients with eating disorders than on MtF patients [3, 5, 6, 8, 9], and the MtF patient in the present study developed eating disorder symptoms after GRS, unlike previous case reports [2, 6]. Lastly, to the best of our knowledge, this is the first study on patients with gender dysphoria and eating disorders from East Asia. Written informed consent was provided for publication by the two patients.

## Case presentation

### CASE 1 (fig. 1)

Case 1 was a 35-year Japanese MtF transsexual patient with bulimia nervosa. Fourteen years had passed since she had undergone GRS and hormonal treatment when she visited our department to seek treatment for her eating disorder. During her early childhood, she already felt that she belonged to the other sex. When she was 12 years, she noticed that her breasts were growing. She was suspected of having a sex development disorder by a pediatrician, but she refused to undergo further examinations. With regard to the fluctuation of her body weight before the GRS, we confirmed that the onset of her eating disorder was not before the GRS. She used to like eating when she was under 10 years old. However, the gradual decrease of her weight was due to the decrease of the amount of her food intake. She said that she had not dieted and she had not cared care much about her body shape and image before the GRS. Therefore, the onset of her eating disorder was after the GRS.

During her adolescence, she had an ongoing gender identity problem. She started taking hormonal treatment using estrogen at the age of 19 years, and several months later she underwent GRS. In addition, she legally changed her first name to a female one at the age of 20 years. At that time, her weight was 80 kg and height 1.71 m (body mass index, BMI, 27.4 kg/m^2^), and binge eating or self-induced vomiting had never occurred to that point in time.

By undergoing GRS, her primary goal should have been achieved. However, she realized that she was not capable of pregnancy and abandoned herself to despair, which led her to binge eating and self-induced vomiting. One of the reasons she was not able to stop these pathological eating behaviors was that she could never disclose her sexual dysphoria, even to her partner, friends, or colleagues. Although her parents knew her situation, she did not want to bother them. When she was not able to stop binge eating, her weight became 100 kg (BMI, 30.9 kg/m^2^), at 27 years. On the other hand, when her self-induced vomiting was dominant, her minimum weight was 57 kg (BMI: 19.5 kg/m^2^), at 30 years. At 30 years, she had a male partner. However, she broke up with him because she was not able to disclose her gender problem and the fact that she was infertile. After she broke up with her partner, she was not able to control binge eating.

She worked as a secretary from 20 to 33 years. However, she had resigned from the job six months before she visited our department for the first time, because she had to spend a lot of time binge eating and she was emotionally unstable due to her fear of becoming fat.

At her first visit to our department at 33 years, her weight was 90 kg (BMI, 30.9 kg/m^2^). Her chief complaint was binge eating and self-induced vomiting once or twice every day. Her symptoms included fear of becoming fat and distorted body image. There was no significant past medical history, psychiatric history, or family history at the time of diagnosis.

She was admitted to the hospital to improve her eating behavior on two different occasions for two weeks each time. Then, she continued to receive outpatient treatment for bulimia nervosa in our department and hormonal treatment by a gynecologist. She said that hormonal treatment was one of the most important things in her life. Although she lives as a woman now receiving GRS and hormonal treatment, she still has episodes of binge eating, self-induced vomiting, and food restriction due to the psychological stress about gender identity.

**Fig. 1:**
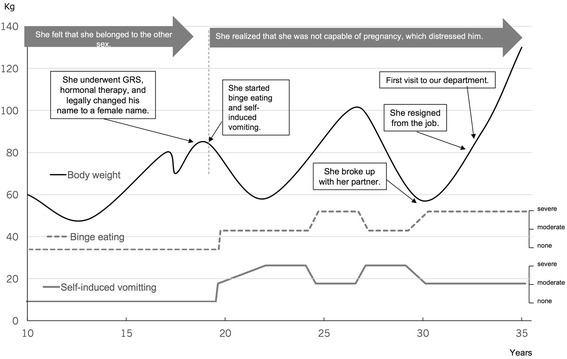
The course of the body weight and eating behavior of the MtF patient. The black line shows body weight change, the dotted line is frequency of self-induced vomiting, and the grey line is frequency of binge eating. She underwent hormonal treatment and GRS. However, she realized that she was infertile, even after GRS, which led her to maintain her eating disorder symptoms and her weight has been fluctuating

Overall, there were three turning points about her eating disorder symptoms (Fig. 1). The first point was after GRS. She realized new gender problems and she started binge eating and self-induced vomiting. The second point was when she broke up with her partner. Then, her binge eating worsened because she could not disclose her gender problem to her partner. The third point was when she resigned from her job due to psychological stress from misunderstandings about gender dysphoria by her colleagues and to exacerbation of binge eating. She was disappointed with the lack of understanding about gender dysphoria in Japan. Although she desired to lower her body weight, she could not help binge eating due to the stress. Therefore, her weight has been fluctuating between 50 and 130 kg.

### CASE 2 (fig. 2)

Case 2 was a 35-year-old Japanese FtM transsexual patient with anorexia nervosa binge eating/purging type. He began hormonal treatment at 24 years and underwent GCS at 27 years; therefore, eight years had passed since he had undergone GCS, when he visited our department to seek treatment for eating disorders.

During his early childhood, he already identified himself with the other sex. When he was 15 years, he desired to stop the development of secondary sexual characteristics. He thought that a lower body weight could prevent breast growth and menstruation. Therefore, he started food restriction, and his body weight decreased to 30 kg, with a height of 1.53 m (BMI, 12.8 kg/m^2^).

He began self-induced vomiting after he entered university to lose weight, but he was able to control his self-induced vomiting until 23 years. Then, he was not able to control his binge eating and his weight increased to 56 kg (BMI, 23.9 kg/m^2^). At 24 years, he disclosed his gender dysphoria and started undergoing hormonal treatment using testosterone. Testosterone prevented the female physical characteristics, and he experienced improvement in his body dissatisfaction, negative feelings, and binge eating. He was also able to increase his body weight. However, three years after starting the hormonal treatment, he restarted self-induced vomiting and his weight decreased. The reason was unclear even after we asked him repeatedly. His doctor suggested that he should discontinue the hormonal treatment due to his low body weight. When he did so, he became emotionally unstable and increased the frequency of self-induced vomiting and binge eating.

Around this time, his body weight decreased to 29 kg (BMI, 12.4 kg/m^2^). He underwent bilateral mastectomy at 27 years, and he legally changed his first name to a male one. Then, he improved his eating behavior and gained weight to 34 kg for one year. When he restarted self-induced vomiting, he had started attending lesbian, gay, bisexual, and transgender (LGBT) community gatherings. He had been distressed about the lack of understanding about gender dysphoria in Japan, but he found LGBT friends with same problems at that time. Therefore, he was able to improve his psychological status and to stop his pathological eating behaviors again, at 30 years. However, he lost weight again due to eating schedule disturbances because his part time job was so busy that he could not have time for lunch, as is often the case for Japanese workers. Then, he visited our department to seek a specialized treatment because he felt muscle weakness.

At his first visit to our department at 31 years, his weight was 29 kg (BMI, 12.4 kg/m^2^). We started self-monitoring of his eating behavior as an outpatient treatment. Then, he was admitted to our inpatient unit because he could not gain weight. During his admission, he was able to increase his weight to 33.4 kg. However, his eating behavior was related to a desire to get rid of his feminine features, and he reported engaging in excessive exercises to increase his muscularity. Therefore, his weight was maintained at approximately 34 kg, and he could not gain any more weight.

His doctor decided to restart hormonal treatment. Subsequently, he achieved improved satisfaction with his appearance, and he became more motivated to gain weight. Finally, he reached a weight of more than 40 kg (BMI, 16.9 kg/m^2^) for the first time in 10 years.

**Fig. 2:**
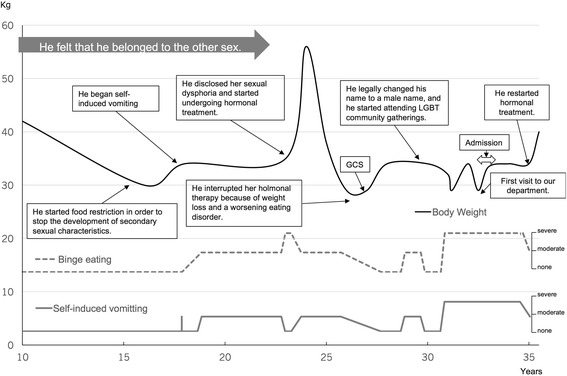
The course of the body weight and eating behavior of the FtM patient. The black line shows body weight change, the dotted line is frequency of self-induced vomiting, and the grey line is frequency of binge eating. He underwent hormonal treatment, and his body dissatisfaction, mood instability, and disordered eating improved. An important turning point was when he joined the LGBT community. He was able to stop binge eating and vomiting for a year. However, he interrupted his therapy because of weight loss and a worsening eating disorder. When he restarted hormonal treatment, he gained weight, and his eating behavior improved again

In summary, there were four turning points in his eating disorder symptoms. The first was related to secondary sexual characteristics. He lost weight to 30 kg to prevent breast growth and menstruation. The second was when he disclosed his gender dysphoria and started undergoing hormonal treatment; the treatment improved his dissatisfaction and he was able to gain weight. However, he began binge eating at that time. The third point was when he legally changed his name and started attending LGBT community gatherings. He was relieved and his eating habits improved. Finally, he restarted hormonal treatment and reached 40 kg (fig. 2).

## Discussion and conclusions

We reported two cases of transsexual persons (one MtF and one FtM) with eating disorders undergoing GRS/GCS. To the best of our knowledge, this study was the first one on the long-term course of eating disorders after GRS/GCS. A previous study reported prolonged anorexia nervosa with FtM [9]; however, they reported for only a few years after hormonal treatment and GCS. Moreover, their case was FtM and he had a good course after treatment. Our first case, a MtF person, had a 14-year-course of bulimia nervosa and Case 2, a FtM person, had a 9-year-course of anorexia nervosa after GRS/GCS, while previous reports described clinical courses of at most a few years after GRS/GCS [2, 4–6, 8]. We found that GRS/GCS did not always influence the course of eating disorders favorably, even after a long period after GRS/GCS.

There are two case reports of MtF persons who first developed eating disorders and then underwent GRS [2, 6]. In contrast, the MtF patient in the present study developed symptoms of eating disorders after GRS. One of those two case reports showed improvement in body satisfaction after GRS, whereas the other did not. However, the long-term course was not clear. Previous studies on people with gender dysphoria and eating disorders reported that GRS could improve the eating behavior [12], body satisfaction, and psychological distress [11, 12]. In contrast, eating disorder symptoms were not improved in the MtF patient in the present study, even after she underwent hormonal treatment and GRS and legally changed her name to a female name. Because she realized that she was infertile and it was hard for her to reveal her gender problem to her partner, she continued to have gender identity problems even after GRS, which led her to maintain her eating disorder symptoms.

In comparison with MtF persons, reports on FtM persons with eating disorders are rare. There have been only five previous case reports on FtM patients with eating disorders [3, 5, 6, 8, 9], and two of the five patients underwent hormonal treatment and GCS [5, 8]. We reported the eating behavior and weight gain patterns of an FtM patient with anorexia nervosa after GCS. After he underwent hormonal treatment, his body dissatisfaction, mood instability, and disordered eating improved, as described in the previous reports. Moreover, we found that joining the LGBT community also improved his symptoms and distress levels.

In addition, many East Asian LGBT persons are distressed by stigma, hostility, and misunderstandings [15, 16]. This study is the first one from East Asia on patients with gender dysphoria and eating disorders. Asian countries emphasize family and social harmony, and sexuality plays a relatively insignificant role in its cultural construction [15]. For example, Asian LGBT people try to conceal their sexual identity by getting married and having children. In addition, LGBT persons have been treated as nonexistent or invisible in Japan [17]. Therefore, people with gender dysphoria could suffer from more psychological stress, which might influence the course of their eating disorders.

In the present study, we considered gender dysphoria as the primary diagnosis. GRS/GCS might reduce body dissatisfaction and uneasiness and improve the eating disorder symptoms of the patients. However, GRS/GCS cannot perfectly change the biological sex, and infertility cannot be resolved by GRS/GCS. Both of our patients, especially case 1, showed that there might remain many gender identity problems, such as fertility, disclosure of their gender dysphoria, and social stigma, which could result in some patients continuing to experience disturbed eating behavior. In addition, identity problems related to gender are also important, and it is difficult to differentiate them from gender identity problems. Therefore, the aspect of identity problems in general should also be considered in the support of patients with gender dysphoria and eating disorders.

In summary, we reported the course of two Japanese patients with gender dysphoria and eating disorders after GRS/GCS, for nine and 14 years, respectively. According to previous reports, GRS/GCS might reduce body dissatisfaction and uneasiness and improve the eating disorder symptoms of patients [11, 12, 14]. However, there are two psychological conflicts for a person with gender dysphoria, core gender identity and to gender role identity [18]. When gender role conflict is persistent, maladaptive eating behaviors could be worsened. In terms of long-term treatment, there might remain many gender role identity problems, such as fertility, disclosure of the gender dysphoria, and social stigma, which could result in some patients continuing to experience disturbed eating behavior. Therefore, continual clinical support should be provided for patients with gender dysphoria and eating disorders, even after hormonal therapy or GRS/GCS, from the viewpoint of gender role identity.

## Abbreviations

ANBP: Anorexia nervosa binge-eating/purging type
BMI: Body mass index
BN: Bulimia nervosa
FtM: Female-to-male
GCS: Gender confirmation surgery
GRS: Gender reassignment surgery
LGBT: Lesbian, gay, bisexual, and transgender
MtF: Male-to-female
